# A Heavy Heart: The Association between Weight and Emotional Words

**DOI:** 10.3389/fpsyg.2016.00920

**Published:** 2016-06-21

**Authors:** Xueru Zhao, Xianyou He, Wei Zhang

**Affiliations:** Guangdong Key Laboratory of Mental Health and Cognitive Science, Center for Studies of Psychological Application, School of Psychology, South China Normal UniversityGuangzhou, China

**Keywords:** weight perception, emotional words, embodied cognition, conceptual metaphor theory, abstract concepts

## Abstract

People often express emotion in language using weight (e.g., a heavy heart, light-hearted, light humor, or heavy-handed), but the question remains whether these expressions of emotion are rooted in the body. Six experiments used a priming paradigm to explore the metaphoric relation between weight perception and emotional words. Experiments 1 and 2 investigated the influence of weight perception on judgments of emotional words and the influence of emotional words on judgments of weight, respectively. A significant difference between the consistent condition (e.g., lightness corresponds to positive words and heaviness corresponds to negative words) and the inconsistent condition (e.g., lightness corresponds to negative words and heaviness corresponds to positive words) was found in Experiment 1 but not in Experiment 2. Experiments 3, 4, and 5 were conducted to exclude potential confounds. Experiment 6 was a repeated-measures study that was conducted to verify the weight-emotion effect. The study confirmed that weight perception affected judgments of emotional words. The results contribute to the growing literature on conceptual metaphor theory and embodied cognition theory.

## Introduction

The way in which an individual represents concepts, especially abstract concepts, has consistently been a core issue in the field of cognitive research. Embodied cognition theory holds that individuals represent abstract concepts through experiences of perception. Lakoff and Johnson (1980, 1999) proposed conceptual metaphor theory to explain how abstract concepts link to perception. They argued that people often refer to familiar, tangible, and concrete concepts to understand unfamiliar, invisible, and abstract concepts. For example, people typically represent abstract words, such as “enthusiasm” and “unconcerned,” using the terms “hot” and “cold,” which reference the sense of temperature. Using conceptual metaphor theory as a basis, Pecher et al. (2011) suggested that image schemas are insufficient to fully represent abstract concepts and that situations or events are also necessary. Furthermore, Borghi and Binkofski (2014) proposed the Words As social Tools (WAT) view, a recent theory regarding embodied cognition and abstract concepts. These authors promulgated the idea that both sensorimotor and linguistic experiences form the basis of abstract concepts and abstract word representation, processing, and use.

Emotions are abstract feelings that are difficult to express. People often use metaphors to express and understand emotions. Emotion is not only an important part of human experience but also a particular psychological phenomenon of human beings. In our daily life, we express our emotions to others and need to read other individuals' emotions to communicate with others more effectively.

Many studies have indicated that words related to emotion can be represented through concrete concepts, such as spatial position, temperature, and color. Meier and Robinson (2004) explored the relationship between emotional words and spatial position. They found that response times were faster and more accurate when positive words were presented at the top of a screen or when negative words were presented at the bottom of a screen. Meier et al. (2004) showed that emotional words and color also have a metaphorical relationship. They reported that positive words were evaluated more quickly when the font color was white rather than black and that negative words were evaluated more quickly when the font color was black rather than white. Meier et al. (2015) replicated the main results of Meier et al. (2004). Other researchers have found that emotion can be represented through temperature (Wilkowski et al., 2009), suggesting that the temperature clue “hot” could contribute to the representation of knowledge related to anger. People typically overestimate the surrounding temperature when priming the emotion stimulus of anger. Du et al. (2013) explored how other emotions, such as sadness, could also be represented by temperature.

Weight is a sense experience in our daily life that can represent many abstract concepts. In language and culture, we can find many words and sentences in which “weight” is related to the concept of importance, for example, “Poor men's words have little weight.” These phrases and words indicate a metaphorical relationship between weight and abstract concepts. People often use “weight,” which can be perceived, to represent “importance,” “power,” and other abstract concepts.

Jostmann et al. (2009) conducted four studies to demonstrate that the abstract concept of importance is grounded in bodily experiences of weight. Participants were asked to hold either a heavy or a light clipboard and were then asked to make judgments of importance in four different situations. The results showed that participants overestimated monetary value and considered fair decision-making procedures more important when they were holding a heavy clipboard.

Ackerman et al. (2010) investigated how metaphorical associations with weight affect impression formation. Participants holding heavy clipboards rated a candidate as better overall and, specifically, as displaying more serious interest in a potential position. This result suggests that the weight cue affected impressions of the candidate's performance and seriousness, consistent with a “heavy” metaphor.

A series of additional studies explored associations between weight and power. Wu et al. (2013) found that responses to weight in an inconsistent condition were slower than those in a neutral condition when power concepts were the prime stimuli. Furthermore, there was a significant difference between the response times of those in the consistent condition and inconsistent condition when weight was used as the prime stimulus. The study indicated that power can be affected by weight and that power can be effectively primed by weight. Lee and Schnall (2014) explored the relation between power and weight with regard to other aspects. Their first study showed that people with a low personal sense of power judged loaded boxes as heavier than people with a high personal sense of power. In Study 2, participants were asked to hold an expansive posture, which could lead to a sense of high power, or to hold a constricted posture, which could lead to a sense of low power. The results showed that participants in the powerless condition perceived the same boxes as heavier. Kouchaki et al. (2014) found that the physical experience of weight is associated with the emotional experience of guilt, and that weight thus intensifies the experience of guilt. For example, participants who wore a heavy backpack experienced higher levels of guilt.

Many experiences of weight occur in daily life. For instance, people carrying heavy objects may feel that a road is more distant and is more difficult to walk compared with those not carrying objects. People may also feel that more time and energy are required to move a heavy object than a light object. Thus, we can conclude that heavy objects require people to expend more energy than light objects. Therefore, people unconsciously associate heavy objects with negative emotion, such as “hardship.”

Many emotional words can be expressed in Chinese through the concept of weight, such as “to be in deep distress,” “to swallow humiliation and bear a heavy load,” and “lightheartedness.” Many similar expressions exist in English, such as “light at heart,” “weight off my shoulders,” and so on. Is emotion rooted in the body in the form of weight? That is to say, does weight have an influence on emotion? The first aim of the current study is to examine these questions.

Some previous research has indicated that perception has a unidirectional effect on the representation of abstract concepts (Casasanto and Boroditsky, 2008), whereas other research has demonstrated a bidirectional relationship between perception and abstract concepts (Giessner and Schubert, 2007). Whether emotion has an effect on people's perception of weight, whether weight affects people's emotions, or whether a mutual influence exists between emotion and weight are unresolved questions. Thus, the second purpose of this paper is to further examine the unidirectional or bidirectional relationship between weight and emotion.

The present study explored the relationship between abstract concepts and body perception from a novel perspective providing new evidence regarding embodied cognition. To address the two questions, we conducted five experiments. These experiments explored the influence of weight perception on judgments of emotional words and the influence of emotional words on judgments of weight. We predicted that participants would perform more quickly in a consistent condition (light weight corresponding to positive words and heavy weight corresponding to negative words) and slower under an inconsistent condition (light weight corresponding to negative words and heavy weight corresponding to positive words), which would indicate that emotion may be rooted in the body in the form of weight. The lack of a significant difference between the consistent condition and the inconsistent condition would indicate that the lack of a metaphorical relation between emotion and weight.

## Experiment 1

### Method

#### Ethics statement

The current study was approved by the Academic Committee of the School of Psychology at South China Normal University. All participants provided written informed consent before participating in the experiments.

#### Participants

In accordance with the participant selection criteria used in prior studies (Richter and Zwaan, 2009; Sherman and Clore, 2009; Thornton et al., 2013), 32 students (10 of whom were male) from South China Normal University participated in Experiment 1. The average age was 22.5 ± 3.74 years. The participants' vision was normal or corrected to normal. Their native language was Chinese, and none of them had a reading disorder. The participants were paid for their participation.

### Materials

**Figure 1:**
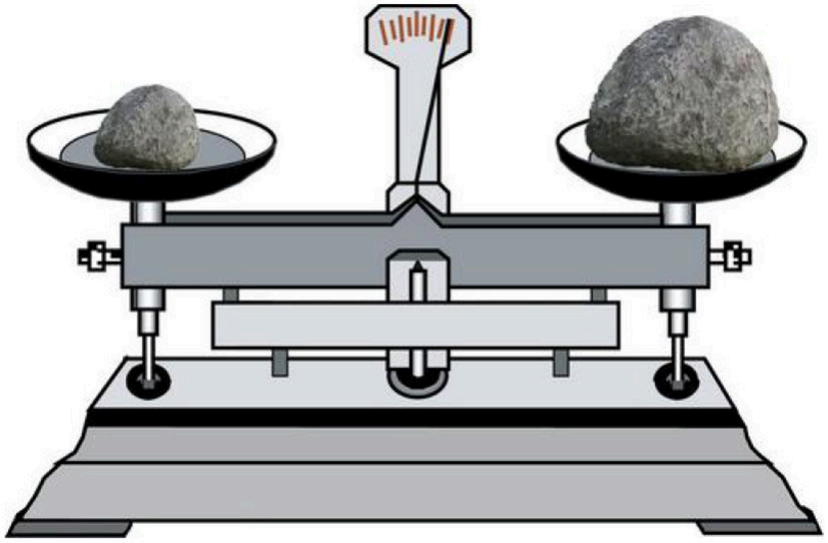
**Example of experimental stimuli (weight)**.

The materials used to represent weight were shown in the form of a balance (see Figure 1). A stone was positioned on the right and the left balance pans. The sizes of the two stones differed, but their color, texture, and density were the same. The balance indicator pointed to the heavier side. That is, the side with the small stone was lighter, and the side with the large stone was heavier. In two cases, either the left side or the right side was heavier.

**Figure 2:**
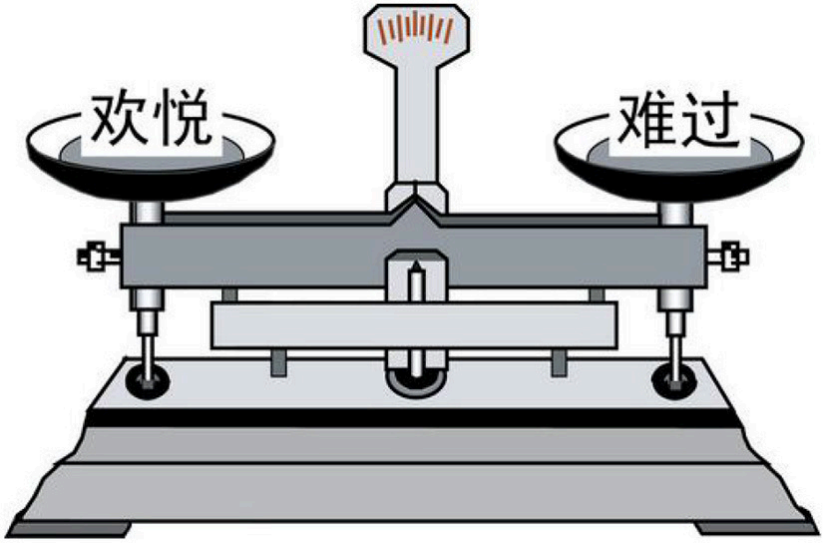
**Examples of experimental stimuli (emotional words)**. The Chinese word “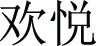 (huan1 yue4)” on the left balance pan means “happy,” whereas “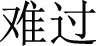 (nan2 guo4)” on the right balance pan means “sad.”

The materials representing emotional words were also presented in the form of a balance (see Figure 2). There was a positive word or a negative word on the right and left balance pans. The positive word and the negative word positioned on the same balance were matched on various aspects, such as emotional valence, familiarity, dominance, and arousal. Most importantly, their stroke numbers were the same. Unlike the weight materials, for the emotional material, the balance did not have a balance indicator. There were 36 positive words and 36 negative words in the formal experiment (see Appendix in Supplementary Material for the materials). Half of the stimuli with positive words appeared on the right balance pan, and the other half appeared on the left balance pan.

Fifteen participants who did not take part in the formal experiments evaluated all of the emotional words in terms of emotional valence, familiarity, dominance, and arousal. All evaluations were made on a 9-point scale. Emotional valence referred to the degree of happiness or sadness, with 1 indicating that the meaning of the emotional word was extremely bad and 9 indicating that the meaning of the emotional word was extremely good. Familiarity referred to the degree of familiarity; 1 indicated that the emotional word was extremely unfamiliar to the participant, and 9 indicated that the emotional word was extremely familiar. Dominance referred to the degree to which one can control the activation of emotional words; 1 indicated that the participant was completely influenced by the emotional word, and 9 indicated that the participant could completely control the activation of the emotional word. Arousal referred to the degree of subjective physiological activation caused by the emotional word, with 1 indicating that the participant was extremely calm and 9 indicating that the participant was extremely excited.

The norming results were as follows. The mean rating of emotional valence was 7.46 ± 0.55 for positive words and 2.55 ± 0.48 for negative words. There was a significant difference between negative words and positive words on the same screen, *F*_(1, 37)_ = 1290.75, *p* < 0.001, η^2^ = 0.972. Regarding familiarity, the mean rating was 8.21 ± 0.40 for positive words and 8.21 ± 0.36 for negative words. We did not find a significant difference between negative words and positive words on the same screen, *F*_(1, 37)_ = 0.003, *p* = 0.955. The mean dominance rating was 5.21 ± 0.42 for positive words and 5.18 ± 0.35 for negative words. No significant difference was found between negative words and positive words on the same screen, *F*_(1, 37)_ = 0.12, *p* = 0.727. In terms of arousal, the mean rating for positive words was 6.53 ± 0.61 and that for negative words was 6.37 ± 0.34. There was no significant difference between negative words and positive words on the same screen, *F*_(1, 37)_ = 1.64, *p* = 0.208.

#### Design

The experimental design included one factor (consistent condition vs. inconsistent condition). In the consistent condition, the light (heavy) weight and positive (negative) words were presented on the same side. In the inconsistent condition, the light (heavy) weight and positive (negative) words were presented on opposite sides. The dependent variable was participants' response time and accuracy.

#### Procedure

**Figure 3:**
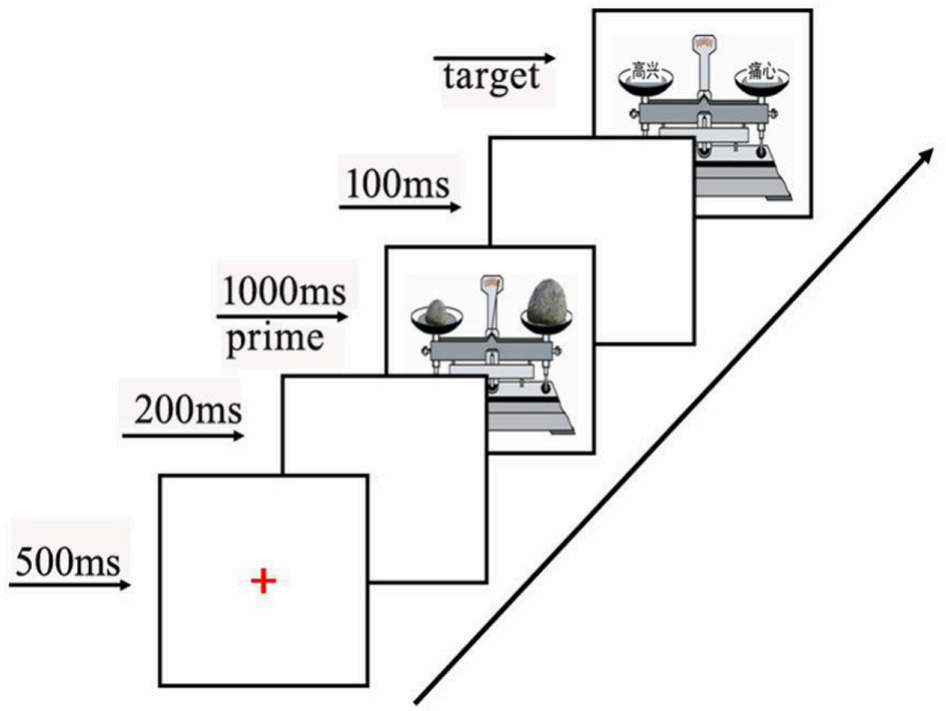
**Example of experimental paradigm**.

Experiment 1 was designed to test whether weight perception affected judgments of emotional words using a priming paradigm. After all instructions were given, the participants began the experiment by pressing the space bar. First, a red fixation cross was presented at the center of the screen for 500 ms. Then, a blank screen was presented for 200 ms. After that, the priming stimulus (the material for weight) was presented for 1000 ms. Another blank screen was shown for 100 ms. Lastly, the target stimulus (the material for emotional words) was presented (see Figure 3). Half of the participants were asked to judge the side on which the positive word was located. The other half of the participants judged the side on which the negative word was located. The target stimulus remained on the screen until a response was given. For half of the priming stimuli with weight, the right side was heavier, and for the other half, the left side was heavier. Each pair of emotional words was shown two times. In one presentation, the positive word was positioned on the right balance pan and the negative word was placed on the left balance pan. In the other presentation, the positive word was on the left balance pan and the negative word was on the right balance pan. The formal experiment included a total of 72 experimental trials.

### Results and discussion

Error responses and Z-scores deviating more than 2 standard deviations from the response mean were excluded. The excluded data represented < 5% of all data. The remaining data were submitted to repeated-measures ANOVAs (see Table 1 for mean response times and accuracy across the different conditions). SPSS 17.0 was used to analyze the data (see Data Sheet in Supplementary Material for all data).

**Table 1:** **Mean response times and accuracy in the consistent and inconsistent conditions in Experiment 1**.

**Condition**	**RT (ms)**	**Accuracy (%)**
Consistent	641.15± 99.04	97.31 ± 3.27
Inconsistent	653.10± 101.00	98.09 ± 2.77

The results indicated a significant difference in response times between the consistent condition and the inconsistent condition [*F*_1(1, 31)_ = 4.56, *p* = 0.041, η^2^ = 0.128; *F*_2(1, 35)_ = 5.33, *p* = 0.027, η^2^ = 0.132]. However, with respect to accuracy, no significant difference was found between the consistent condition and the inconsistent condition [*F*_1(1, 31)_ = 1.39, *p* = 0.248; *F*_2(1, 35)_ = 1.84, *p* = 0.183].

These findings indicate that the participants' perception of weight affected their judgments of emotional words. Their response time was faster when the light (heavy) weight and positive (negative) words were positioned on the same side. When they were not presented on the same side, the participants' response times were longer. However, no significant difference was found in the analysis of accuracy data. Furthermore, although there was a significant difference in response time, the difference in the average response time was only ~12 s between the consistent and inconsistent conditions. One potential explanation for this finding is that the participants were highly familiar with the emotional words used as experimental materials (i.e., mean familiarity was 8.21 in the norming study). Strong familiarity may have facilitated both accuracy and response times, reducing the differences between the two conditions.

## Experiment 2

### Method

#### Ethics statement

Please see the ethics statement provided for Experiment 1.

#### Participants

Thirty-two students (10 males) from South China Normal University participated in Experiment 2. The average age was 21.5 ± 1.27 years. The criteria used to select the participants were the same as those employed in Experiment 1.

### Materials

The materials were the same as those used in Experiment 1.

#### Design

The design was the same as that employed in Experiment 1.

#### Procedure

In Experiment 2, a priming paradigm was used to test whether emotional words affect judgments of weight. In Experiment 2, the priming stimuli were emotional words, and the target stimuli were weights. Half of the participants were asked to judge which side of the balance was lighter, and the other half judged which side was heavier. The remainder of the procedure was the same as that utilized in Experiment 1.

### Results and discussion

The RTs of correct responses and the accuracy data were submitted to analysis. The same outlier procedure used in Experiment 1 was performed, reducing the data set by < 5%. The same data analyses employed in Experiment 1 were conducted (see Table 2 for mean response times and accuracy across the different conditions).

**Table 2:** **Mean response times and accuracy in the consistent and inconsistent conditions in Experiment 2**.

**Condition**	**RT (ms)**	**Accuracy (%)**
Consistent	425.01 ± 52.42	99.57 ± 1.24
Inconsistent	427.66 ± 54.93	99.74 ± 0.82

The results showed no significant difference in either response time [*F*_1(1, 31)_ = 0.97, *p* = 0.331; *F*_2(1, 35)_ = 0.46, *p* = 0.501] or accuracy [*F*_1(1, 31)_ = 1.00, *p* = 0.325, *F*_2(1, 35)_ = 0.66, *p* = 0.422] between the consistent condition and the inconsistent condition. This result indicated that emotional words had no influence on the participants' perception of weight.

However, the materials used to represent weight in Experiments 1 and 2 are problematic. A small or large stone was positioned on the right or left balance pan, respectively. Experiment 1 found that weight perception significantly affected judgments of emotional words. Did the weight of the stone or the size of the stone produce this significant difference? Experiment 3 was designed to consider this possibility.

## Experiment 3

### Method

#### Ethics statement

Please see the ethics statement listed for Experiment 1.

#### Participants

Thirty students (8 males) from South China Normal University took part in Experiment 3. The average age was 22.67 ± 2.19 years. Participants were selected based on the same criteria considered in Experiment 1.

### Materials

**Figure 4:**
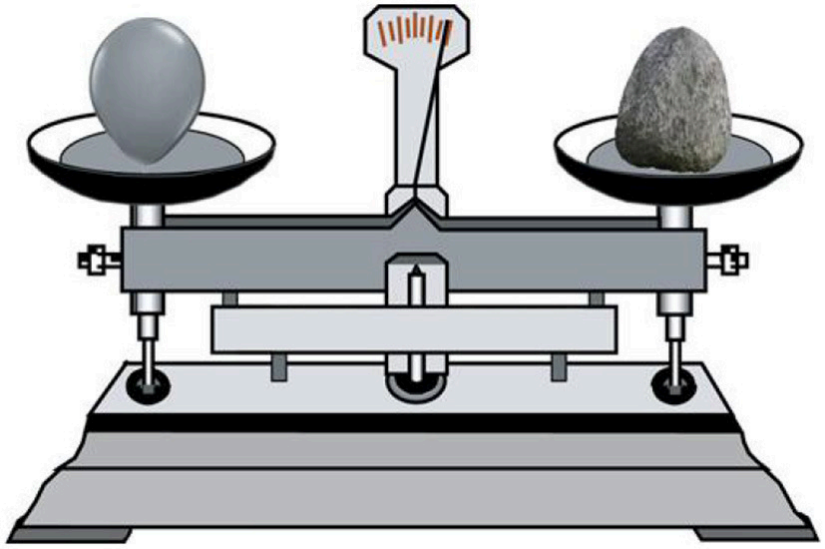
**Example of experimental stimuli (weight) in Experiment 3**.

As in Experiments 1 and 2, the materials used to display weight were shown on a balance. However, a balloon and a stone of the same size replaced a large stone and a small stone on the balance pans (as shown in Figure 4). The emotional words were the same as those utilized in Experiment 1.

#### Design

The design was the same as that employed in Experiment 1.

#### Procedure

The procedure was the same as that followed in Experiment 1.

### Results and discussion

The RTs of correct responses and the accuracy data were analyzed. The same outlier procedure employed in Experiment 1 was conducted, reducing the data set by < 5%. The same data analyses performed in Experiment 1 were conducted (see Table 3 for mean response times and accuracy across the different conditions).

**Table 3:** **Mean response times and accuracy in the consistent and inconsistent conditions in Experiment 3**.

**Condition**	**RT (ms)**	**Accuracy (%)**
Consistent	633.09 ± 117.73	96.76 ± 3.87
Inconsistent	646.52 ± 123.11	97.41 ± 3.09

The results showed a significant difference in response time between the consistent condition and the inconsistent condition [*F*_1(1, 29)_ = 7.88, *p* = 0.009, η^2^ = 0.214; *F*_2(1, 35)_ = 5.67, *p* = 0.023, η^2^ = 0.139]. Similar to the findings of Experiment 1, these results indicate no significant difference in accuracy [*F*_1(1, 29)_ = 0.96, *p* = 0.335; *F*_2(1, 35)_ = 0.77, *p* = 0.385] between the consistent condition and the inconsistent condition.

When the confusing variable (i.e., the size of the stimuli) was controlled, the significant difference in response time between the consistent condition and the inconsistent condition remained. Therefore, we can conclude that the participants in Experiment 1 were affected by the weight of the two stones on the balance pan rather than by the size of these two stones. Hence, the participants' perceptions of weight affected their judgments of emotional words.

However, the results may still be confounded by other factors. One concern regarding the materials used in Experiment 3 regarded the shape of the objects. Although the balloon and the stone were the same size, the balloon had a narrow bottom and a wide top, whereas the stone had a wide bottom and a narrow top. In addition, a balance pointer was located on the balance in the weight materials to highlight the light and heavy weights of the objects. As such, participants may have been affected by the spatial direction of the balance pointer rather than the weight of the object. Furthermore, the presentation time on the blank screen was fixed; thus, participants could easily form a fixed expectation. We designed Experiments 4 and 5 to resolve these potential confounds.

## Experiment 4

### Method

#### Ethics statement

Please refer to the ethics statement provided for Experiment 1.

#### Participants

Thirty students (8 males) from South China Normal University took part in Experiment 3. The average age was 20.5 ± 1.78 years. The criteria used to select the participants were the same as those employed in Experiment 1.

### Materials

**Figure 5:**
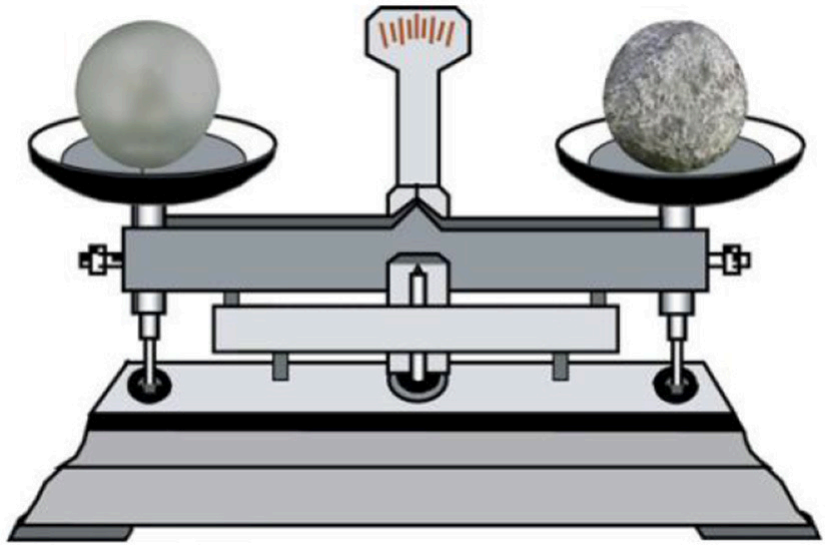
**Example of experimental stimuli (weight) in Experiment 4**.

The materials used to represent weight in Experiment 4 were improved based on the materials used to represent weight in Experiment 3. The balloon and the stone were made the same shape, and the balance pointer was removed (as shown in Figure 5). The emotional words were the same as those in Experiment 1.

#### Design

The design was the same as that used in Experiment 1.

#### Procedure

The blank screen was presented for 100, 300, and 500 ms randomly. The remainder of the procedure was the same as that followed in Experiment 1.

### Results and discussion

The RTs of correct responses and the accuracy data were submitted to analysis. The same outlier procedure performed in Experiment 1 was conducted, reducing the data set by < 5%. The data analyses conducted in Experiment 1 were also conducted for this experiment (see Table 4 for mean response times and accuracy across the different conditions).

**Table 4:** **Mean response times and accuracy in the consistent and inconsistent conditions in Experiment 4**.

**Condition**	**RT (ms)**	**Accuracy (%)**
Consistent	610.82 ± 119.68	97.50 ± 2.86
Inconsistent	623.54 ± 112.20	98.05 ± 2.54

The results showed that for the analysis of response time data, there was a significant difference between the consistent condition and the inconsistent condition in the by-participant analysis [*F*_1(1, 29)_ = 5.89, *p* = 0.022, η^2^ = 0.169]. Furthermore, a marginally significant difference was found in the by-item analysis [*F*_2(1, 35)_ = 3.55, *p* = 0.068, η^2^ = 0.092]. Similar to the findings of the prior experiments, no significant difference in accuracy was found between the consistent condition and the inconsistent condition [*F*_1(1, 29)_ = 0.78, *p* = 0.386; *F*_2(1, 35)_ = 0.52, *p* = 0.475].

When the experimental materials representing weight were further controlled and the time of the blank screen was presented randomly, the participants' reactions in the consistent condition were still faster than that of participants in the inconsistent condition. Experiment 5 was conducted to determine whether emotional words affect judgments of weight when the experimental materials used to represent weight were further controlled.

## Experiment 5

### Method

#### Ethics statement

Please refer to the ethics statement presented for Experiment 1.

#### Participants

Thirty students (8 males) from South China Normal University participated in Experiment 5. The participants' average age was 20.33 ± 1.52 years. Participants were selected based on the same criteria employed in Experiment 1.

### Materials

The materials representing weight were the same as those used in Experiment 4. The materials used to represent emotional words were the same as those employed in Experiment 1.

#### Design

The design used in Experiment 1 was also used here.

#### Procedure

Experiment 5 was designed to test whether emotional words affected judgments of weight using a priming paradigm. The priming stimuli were emotional words, and the target stimuli were weights, as in Experiment 2. Half of the participants were asked to judge which side of the balance was lighter, and the other half of the participants were asked to judge which side was heavier. The remainder of the procedure was the same as that employed in Experiment 4.

### Results and discussion

The RTs of correct responses and the accuracy data were analyzed. We conducted the same outlier procedure as that used in Experiment 1, reducing the data set by < 5%. The same data analyses as those performed in Experiment 1 were conducted (see Table 5 for mean response times and accuracy across the different conditions).

**Table 5:** **Mean response times and accuracy in the consistent and inconsistent conditions in Experiment 5**.

**Condition**	**RT (ms)**	**Accuracy (%)**
Consistent	423.25 ± 70.29	99.72 ± 0.85
Inconsistent	426.13 ± 73.70	99.81 ± 0.70

The results showed no significant difference in either response time [*F*_1(1, 29)_ = 1.19, *p* = 0.285; *F*_2(1, 35)_ = 0.13, *p* = 0.720] or accuracy [*F*_1(1, 29)_ = 0.20, *p* = 0.662; *F*_2(1, 35)_ = 0.33, *p* = 0.571] between the consistent condition and the inconsistent condition.

An observation from this experiment was that emotional words had no significant effect on judgments of weight. Although a slight difference was found between the means of the consistent condition and those of the inconsistent condition, the difference did not reach a significant level.

Although significant differences between the two conditions were found in Experiments 1, 3, and 4, the difference in the average response time was small. Hence, we replicated Experiment 1 to explore the potential weight-emotion relation.

## Experiment 6

### Method

#### Ethics statement

Please see the ethics statement listed for Experiment 1.

#### Participants

Thirty-two students (8 males) from South China Normal University took part in Experiment 6. The average age was 20.50 ± 1.90 years. The participants were selected based on the same criteria followed in Experiment 1.

### Materials

The materials were the same as those used in Experiment 1.

#### Design

The design was the same as that employed in Experiment 1.

#### Procedure

The same procedure used in Experiment 1 was performed here.

### Results and discussion

The RTs of correct responses and the accuracy data were submitted to analysis. The outlier procedure followed in Experiment 1 was also conducted for this experiment, reducing the data set by < 5%. The same data analyses performed in Experiment 1 were conducted (see Table 6 for mean response times and accuracy across the different conditions).

**Table 6:** **Mean response times and accuracy in the consistent and inconsistent conditions in Experiment 6**.

**Condition**	**RT (ms)**	**Accuracy (%)**
Consistent	577.28 ± 88.05	96.70 ± 2.94
Inconsistent	588.75 ± 87.98	96.18 ± 4.10

The results demonstrated a significant difference in response time between the consistent condition and the inconsistent condition [*F*_1(1, 31)_ = 6.36, *p* = 0.017, η^2^ = 0.170; *F*_2(1, 35)_ = 6.52, *p* = 0.015, η^2^ = 0.157]. No significant difference in accuracy [*F*_1(1, 31)_ = 0.78, *p* = 0.385; *F*_2(1, 35)_ = 0.30, *p* = 0.585] was found between the two conditions. The results indicated that the perception of weight had an effect on emotional words, although the difference in mean response time was small.

## General discussion

In the present study we conducted six experiments and confirmed that weight perception affected judgments of emotional words, further enriching theories of embodied cognition. Why is there a metaphoric relationship between emotion and weight? This relation may be derived from the body's experience. Generally speaking, people's perception of weight can be divided into two aspects: the feeling of weight on the body and the weight of psychological pressure. Both of these aspects can cause an emotional change. For instance, a person may feel tired when holding heavy things, which would reinforce a negative mood. Alternatively, a person may feel sad and unhappy when his/her psychological burden is extremely heavy. This situation may lead to the effect of “a heavy heart.”

Of note, none of the emotional words concern “weight.” First, none of the emotional words used in the experiments include the character “
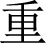
” (heavy) or “
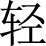
” (light) directly. In addition, the meaning of the words that were used does not relate to weight. For example, the following words were used as materials: 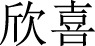 (xin1 xi3, “glad”), 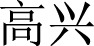 (gao1 xing4, “happy”), 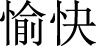 (yu2 kuai4, “joyful”), 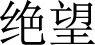 (jue2 wang4, “despair”), 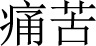 (tong4 ku3, “misery”), 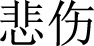 (bei1 shang1, “ sad”). They are not related to weight (heaviness or lightness). Hence, there is no semantic connection between the emotional words and stones because emotional words do directly indicate weight; thus, the weight-emotion effect was not caused by semantic associations.

Lakoff and Johnson (1980) posited that conceptual metaphors are unidirectional. For example, people often use the physical temperature that they feel to perceive warmth and coldness in interpersonal relationships. However, the abstract concept of warmth and coldness in interpersonal relationships does not affect people's perception of the physical temperature. Similarly, Boroditsky (2000) found that the priming of space influenced people's evaluation of time, but related information about time did not affect perception of space.

In recent years, empirical studies have increasingly shown that cognitive processes and sensorimotor processes are interrelated and interact with each other (Barsalou, 2008; Wu et al., 2011). Giessner and Schubert (2007) explored whether judgments of leaders' power and information in a vertical location were interrelated. They found that a longer vertical line increased the judgment of power. On the contrary, a leader's power influenced the participants' vertical positioning of the leader's box in an organization chart and of the leader's picture in a team picture. Some studies have shown that tall men are more likely to obtain a high salary and a higher professional status and to become leaders (Stogdill, 1948; Young and French, 1996; Judge and Cable, 2004). However, other studies asked participants to judge the height of individuals who had different levels of authority or academic status and showed that people with high power and high academic status were considered taller (Dannenmaier and Thumin, 1964; Wilson, 1968). Williams and Bargh (2008) found that individuals were likely to evaluate others more positively and to perform altruistic behaviors if they felt warm. Zhong and Leonardelli (2008) asked participants to recall the experience of being rejected by others and then asked them to estimate the physical temperature of the laboratory. The results showed that participants underestimated the physical temperature when they recalled experiences of being neglected by others.

Is there a unidirectional or bidirectional relationship between perception and abstract stimuli? No unanimous conclusion can be drawn from previous studies. The present study found that although people's weight perceptions affected their judgments of emotional words, emotional words had no influence on judgments of weight. This finding supports the idea that perception has a unidirectional effect on the representation of abstract concepts in certain metaphorical relationships. Why does weight perception affect judgments of emotional words but not vice versa? The reason may be as follows. On the one hand, the notion that weight affects emotion is well established and is strengthened in daily life. People who experience physical or mental weight may have negative moods. On the other hand, emotion, as an abstract concept, may be rooted in the body in many forms, such as space, color, and temperature. Weight is only one of the forms in which emotion can be grounded in the body. As a result, emotional words do not affect people's perceptions of weight. These results are consistent with Firestone and Scholl's (2015) suggestion that cognition does not have top-down effects on perception.

However, some research has demonstrated that emotional stimuli may affect perception. Anelli et al. (2013) found a dynamic affordance effect elicited by neutral objects and an escape-avoidance effect provoked by dangerous objects in a dynamic situation; these findings indicated that perception was influenced by the presence of threatening stimuli. Fantoni et al. (2016) also demonstrated that an observer's internal states induced by bodily actions can affect perception. With respect to how emotion influences perception, Stefanucci et al. (2011) suggested that emotions (e.g., fear, disgust, and sadness) can produce changes in vision and audition and that the perceptual system may be highly interconnected. Thus, emotional information may influence perception in some circumstances.

Given the above discussion, another possible explanation for our results exists. Specifically, emotion does not affect weight because of the ease of judging which side is heavier or lighter. In all cases, response times regarding weights were ~400 ms; therefore, approaching a ceiling effect. Hence, no significant difference was found between the consistent condition and the inconsistent condition when the participants were asked to judge weight. Thus, response time may not be a good indicator to employ when exploring this question. Further studies might better elucidate potential effects of emotion on weight judgments by using brain imaging techniques.

Nonetheless, additional concerns regard individual and potential cultural differences, and the lack of consensus across studies about weight. For example, Schneider et al. (2011) demonstrated that the importance of the information contained in a book caused participants to perceive the book as heavier, providing some evidence that the judgments of importance affect weight perception. Similarly, Schneider et al. (2014) found that a data storage device that holds important tax information or personally relevant information is estimated to be heavier than a similar device without such information. Although some studies have found a metaphorical relationship between weight and importance, others have not. For example, Rabelo et al. (2015) found that weight had no statistically significant effects on judgments of importance across three studies. Based on a meta-analysis of published studies, they also suggested that there is no convincing support for the weight-importance effect. Jostmann et al. (in press) also concluded that no reliable evidence for the effect exists. One possible explanation for these contradictory findings is that such an effect exists only in some cultures (among some people) and not in other cultures (among other people). If a person never or rarely experiences the association between weight and importance (or other metaphorical relationships), he/she may not form the weight-importance connection. Even when an individual experiences the weight-importance association, this effect may not be strongly grounded in the body.

In conclusion, large individual differences in this effect are evident. Moreover, the methods that most previous weight-importance studies used were all subjective assessments, which are affected by individual subjectivity. The present study explored the weight-emotion association using a more objective method (RT as one of the indices). Hence, the results are less impacted by individual subjectivity. To solve these problems, future studies need to improve conceptual metaphor theory and embodied cognition theory or propose a new theory to explain these contradictory findings. Furthermore, a more effective and accurate method (for example, a cognitive neuroscience research technique) must be developed.

## Author contributions

XZ and XH designed the research. XZ and WZ conducted the research. XZ analyzed the data. XZ and XH wrote the manuscript. All authors approved the final version of the manuscript for submission.

### Conflict of interest statement

The authors declare that the research was conducted in the absence of any commercial or financial relationships that could be construed as a potential conflict of interest.
