# Supplementary material for: A Heavy Heart: The Association between Weight and Emotional Words
Source: Front Psychol. 2016 Jun 21;7:920. doi: 10.3389/fpsyg.2016.00920 (PMC4914497; doi:10.3389/fpsyg.2016.00920)
Supplement: Supplementary file 2 [file DataSheet2.pdf]

Appendix

The emotional words used in all five experiments.

自信(zi4 xin4, "self-confidence")	振奋(zhen4 fen4, "hearten")	欣喜(xin1 xi3, "glad")	惊喜(jing1 xi4, "a pleasant surprise")
高兴(gao1 xing4, "happy")	欢呼(huan1 hu1, "cheer")	愉快(yu2 kuai4, "joyful")	安康(an1 kang1, "well-being")
雀跃(que4 yue4, "jump for joy")	惬意(qie4 yi4, "pleasant")	嬉笑（xi1 xiao4, "fun"）	欢喜(huan1 xi3, "gladness")
欣慰(xin1 wei4, "be relieved")	畅快(chang4 kuai4, "delighted")	称心(chen4 xin1, "be gratified")	活泼(huo2 po1, "lively")
大喜(da4 xi3, "exultancy")	欢腾(huan1 teng2, "jubilation")	怡然(yi2 ran2, "satisfied and happy")	欢快(huan1 kuai4, "in a merry mood")
欢悦(huan1 yue4, "pleased")	大笑(da4 xiao4, "laughter")	开朗(kai1 lang3, "optimistic")	快乐(kuai4 le4, "happy")
快活(kuai4 huo2, "merry")	幸福(xi4 fu2, "happiness")	愉悦(yu2 yue4, "joyful")	微笑(wei1 xiao4, "smile")
乐观(le4 guan1, "optimistic")	喜悦(xi3 yue4, "gladness")	舒畅(shu1 chang4, "happy")	兴奋(xing1 fen4, "exciting")
舒心(shu1 xin1, "be pleased")	欢乐(huan1 le4, "happy")	自豪(zi4 hao2, "be proud of")	舒适(shu1 shi4, "comfortable")
哀伤(ai1 shang1, "sad")	难受(nan2 shou4, "unhappy")	绝望(jue2 wang4, "despair")	悲惨(bei1 can3, "miserable")
痛心(tong4 xin1, "distressed")	哀叹(ai1 tan4, "lament")	阴暗(yin1 an4, "gloomy")	忧虑(you1 lv4, "worried")
悲凉(bei1 liang2, "dismal")	悲伤(bei1 shang1, "sad")	痛楚(tong4 chu3, "suffering")	悲痛(bei1 tong4, "grieved")
惭愧(can2 kui4, "ashamed")	苦闷(ku3 men4, "depressed")	纠结(jiu1 jie2, "tangled")	孤独(gu1 du4, "lonely")
抑郁(yi4 yu4, "depressed")	伤感(shang1 gan3, "sad")	悲哀(bei1 ai1, "grief")	忧愁(you1 chou2, "worried")
愁闷(chou2 men4, "feel gloomy")	忧伤(you1 shang1, "distressed")	压抑(ya1 yi4, "depressed")	难过(nan2 guo4, "sad")
沮丧(ju3 sang4, "dispirited")	哀痛(ai1 tong4, "grief")	自卑(zi4 bei1, "self-contempt")	内疚(nei4 jiu4, "guilt")
忧心(you1 xin1, "worried")	哀愁(ai1 chou2, "sad")	疲惫(pi2 bei4, "weary")	凄楚(qi1 chu3, "miserable")
失望(shi1 wang4, "despair")	痛苦(tong4 ku3, "misery")	不快(bu4 kuai4, "unhappy")	阴郁(yin1 yu4, "gloomy")
